# Plant Extract of *Limonium gmelinii* Attenuates Oxidative Responses in Neurons, Astrocytes, and Cerebral Endothelial Cells In Vitro and Improves Motor Functions of Rats after Middle Cerebral Artery Occlusion

**DOI:** 10.3390/antiox10111814

**Published:** 2021-11-15

**Authors:** Tulendy Nurkenov, Andrey Tsoy, Farkhad Olzhayev, Elvira Abzhanova, Anel Turgambayeva, Aizhan Zhussupova, Bharathi Avula, Samir Ross, Aigerim Aituarova, Dariya Kassymova, Galiya Zhusupova, Tamara Shalakhmetova, Tursonjan Tokay, James C. Lee, Sholpan Askarova

**Affiliations:** 1 Faculty of Biology and Biotechnology, Al-Farabi Kazakh National University, Almaty 050040, Kazakhstan; t.nurkenov@abaiuniversity.edu.kz (T.N.); Aizhan.Zhussupova@kaznu.kz (A.Z.); aytuarova.a@mail.ru (A.A.); darik459@gmail.com (D.K.); Galiya.Zhusupova@kaznu.kz (G.Z.); Tamara.Shalakhmetova@kaznu.kz (T.S.); 2 Department of Biology, Institute of Natural Science and Geography, Abai Kazakh National Pedagogical University, Almaty 050010, Kazakhstan; 3 National Laboratory Astana, Nazarbayev University, Nur-Sultan 010000, Kazakhstan; andrey.tsoy@nu.edu.kz (A.T.); folzhayev@nu.edu.kz (F.O.); elvira.abzhanova@nu.edu.kz (E.A.); anel.turgambayeva@alumni.nu.edu.kz (A.T.); 4 School of Pharmacy, University of Mississippi, Oxford, MS 38677, USA; bavula@olemiss.edu (B.A.); sross@olemiss.edu (S.R.); 5 School of Sciences and Humanities, Nazarbayev University, Nur-Sultan 010000, Kazakhstan; tursonjan.tokay@nu.edu.kz; 6 Department of Biomedical Engineering, University of Illinois at Chicago, Chicago, IL 60612, USA; leejam@uic.edu

**Keywords:** antioxidants, neurons, astrocytes, CECs, *L. gmelinii*, plant polyphenols

## Abstract

There are numerous publications demonstrating that plant polyphenols can reduce oxidative stress and inflammatory processes in the brain. In the present study we have investigated the neuroprotective effect of plant extract isolated from the roots of *L. gmelinii* since it contains a rich source of polyphenols and other biologically active compounds. We have applied an oxidative and inflammatory model induced by NMDA, H_2_O_2_, and TNF-α in human primary neurons and astrocytes, and mouse cerebral endothelial cell (CECs) line in vitro. The levels of ROS generation, NADPH oxidase activation, P-selectin expression, and activity of ERK1/2 were evaluated by quantitative immunofluorescence analysis, confocal microscopy, and MAPK assay. In vivo, sensorimotor functions in rats with middle cerebral artery occlusion (MCAO) were assessed. In neurons NMDA induced overproduction of ROS, in astrocytes TNF-α initiated ROS generation, NADPH oxidase activation, and phosphorylation of ERK1/2. In CECs, the exposure by TNF-α induced oxidative stress and triggered the accumulation of P-selectin on the surface of the cells. In turn, pre-treatment of the cells with the extract of *L. gmelinii* suppressed oxidative stress in all cell types and pro-inflammatory responses in astrocytes and CECs. In vivo, the treatment with *L. gmelinii* extract improved motor activity in rats with MCAO.

## 1. Introduction

Oxidative stress is induced by an imbalance of the redox system due to the overproduction of reactive oxygen species (ROS) and/or the disturbance of the antioxidant systems. The brain is an organ that is especially susceptible to the effects of ROS due to its high oxygen demand, increased levels of polyunsaturated fatty acids in neuronal cells, and relatively low levels of antioxidant enzymes [1]. ROS damage lipids, proteins, DNA, RNA, and alter signaling pathways leading to necrotic and apoptotic neuronal death [2]. Additionally, increased ROS stimulates cerebral endothelial cells and astrocytes to secrete inflammatory cytokines and chemokines, and causes the upregulation of adhesion molecules in the cerebral vasculature and the recruitment of peripheral leukocytes [3,4]. The deployment of immune cells is primarily designed to tackle pathogens, and their activation during the sterile immune response further destroys nerve tissue [5]. Previous studies have demonstrated that oxidative stress is one of the leading pathophysiological reactions participating in the development and progression of neurodegenerative diseases such as Alzheimer’s disease and Parkinson’s disease [1], and it also plays a central role in ischemic brain injury [5]. Thus, agents targeting ROS production, activation of pro-inflammatory cytokines, and expression of adhesion molecules have therapeutic value in brain disorder therapy.

As oxidative stress is one of the universal mechanisms of different pathological conditions in the brain, antioxidants have been considered as therapeutics and preventive measures to inhibit neuroinflammation and detrimental impacts of ROS [6]. There are lots of compounds that have prominent antioxidant and anti-inflammatory potential. Compounds such as phenolic acids, proanthocyanidins, and flavonoids are attracting great interest because of their ability to scavenge free radicals and inhibit oxidative damage. Nowadays, plant polyphenols occupy a unique place in medicine as they are potentially capable of reducing the risk of many diseases and might be used in the treatment of diabetes, cardiovascular disorders, atherosclerosis, neurodegenerative disorders, and inflammation.

Polyphenols are natural organic molecules consisting of a huge number of phenol structural units. They are commonly presented in vegetables, fruits, grains, bark, roots, tea, and wine. Plant polyphenols possess many unique physical, chemical, and biological (metabolic, toxic, therapeutic, etc.) properties directly dependent on the number and characteristics of phenol units and their structure. Most polyphenols are generally known to possess potent antioxidant, anti-inflammatory, and anti-apoptotic properties, and their neuroprotective effects have been demonstrated in several in vitro and in vivo studies [7,8,9,10]. For example, magnolia polyphenols have been shown to attenuate oxidative and inflammatory responses in neurons and microglia cells [8]. Green tea polyphenols have also been proven to exhibit multiple neuroprotective actions [11,12]. However, the health effects of polyphenols depend on the chemical structure (e.g., glycosylation, esterification, and polymerization) and bioavailability. Bioavailability appears to differ greatly between the various polyphenols, and the most abundant polyphenols in our diet are not those that have the best bioavailability profile [13]. Thus, new plant sources rich with bioavailable polyphenols are still in demand.

We have previously reported that the plant substance extracted from roots of *Limonium gmelinii* (*Plumbaginaceae*) possesses significant anti-oxidative and hepatoprotective activities, exceeding those of single flavonoids [14]. This plant is a rich source of flavonols and their glycosides, phenolic acids, monomeric, dimeric, and oligomeric forms of flavan-3-ols; it also contains other biologically active compounds, including all known 20 natural α-amino acids, vitamins C, E, and ß-carotene. *L. gmelinii* is widespread in Eastern Europe, South-Western Siberia, China, and Central Asia [15]. It has industrial resources on the whole territory of Kazakhstan, primarily on saline lands, and has been used in traditional herbal medicine in Central Asia for hundreds of years. These plants are characterized by rapid growth and high yield, so their reserves in nature can be kept at their original level if the guidelines of good practices for collection and preparation of medicinal plant raw materials are followed. These plants can also be cultivated. In the present study, we have assessed antioxidant and anti-inflammatory properties of plant extract from *Limonium gmelinii* in neurons, astrocytes, and cerebral endothelial cells (CECs) in vitro, and evaluated its therapeutic potential on animal models of ischemic brain injury. Our choice of cells was dictated by the fact that, as mentioned before, neurons are highly susceptible to oxidative stress [1]. In turn, increasing evidence exists, demonstrating that both astrocytes and cerebral endothelial cells are intensively involved in different aspects of CNS homeostasis including oxidative stress regulation, and the redox level in CNS can also affect astrocytes and CECs in morphology and function [3,4]. In our study, we have applied human primary neurons and astrocytes. We have chosen mouse bEnd3 cerebral endothelial cell line (ATCC) as a model of human brain cerebral endothelial cells since we have previously reported that this cell line responds to the different treatments in a similar way to human primary CECs [16].

## 2. Materials and Methods

### 2.1. Preparation and Chemical Characterization of L. gmelinii Roots Extract Using Liquid Chromatography Diode Array Detector-Quadrupole Time-of-Flight Mass Spectrometry (LC-DAD-QToF)

Preparation of the substance from the roots of *L. gmelinii* was carried out according to a simple, economically, and ecologically viable technological scheme with a high yield output equal to 30–40% of the weight of the dried and processed raw material. The substance was obtained by double extraction of the plant material with 50% ethanol at a feed-solvent ratio of 1:6 and a one-time extraction time of 5 h. The combined filtered extracts were concentrated in a vacuum to dryness at 40–60 °C to get the desired product [17]. The substance is a brownish powder of bitter-astringent taste with a weak specific odor. It is insoluble in hydrophobic solvents, soluble in hydrophilic solvents, such as dimethyl sulfoxide, dimethylformamide, in aqueous ethanol solutions (30% and 50%), moderately soluble in water at 25 °C, soluble at 40–50 °C and highly soluble in water at 100 °C.

A total of 50 mg of extract was weighed and sonicated in 1.0 mL of methanol for 15 min followed by centrifugation for 10 min at 9000 rpm. The supernatant clear solution was used for analysis. The liquid chromatographic system was an Agilent 1200 Series Rapid Resolution LC system, and the separation was achieved on an Agilent Poroshell 120 EC-C18 (150 × 2.1 mm, 2.7 µ) column. The mobile phase consisted of water with 0.1% formic acid (A) and acetonitrile with 0.1% formic acid (B) at a flow rate of 0.35 mL/min. Analysis was performed using the following gradient elution: 95% A/5% B to 70% A/30% B in 15 min and in next 10 min to 70% B. Each run was followed by a 5 min wash with 100% B and an equilibration period of 5 min with 95% A/5% B. Each run was followed by a 5 min wash with 100% B and an equilibration period of 5 min with 65% A/35% B. Two microliters of the sample were injected. The column temperature was 40 °C.

The mass spectrometric analysis was performed with a QToF-MS-MS (Model #G6530A, Agilent Technologies, Santa Clara, CA, USA) equipped with an ESI source with Jet Stream technology using the following parameters: drying gas (N_2_) flow rate, 11.0 L/min; drying gas temperature, 275 °C; nebulizer pressure, 25 psi, sheath gas temperature, 325 °C; sheath gas flow, 11 L/min; capillary voltage, 3000 V; skimmer, 65 V; Oct RFV, 750 V; and fragmentor voltage, 150 V. All the operations, acquisition and analysis of data were controlled by Agilent MassHunter Data Acquisition Software Ver. A.05.01 and processed with MassHunter Qualitative Analysis B.07.00 Service Pack. Each sample was analyzed in positive mode over the range of *m*/*z* = 100–1100 and extended dynamic range (flight time to *m*/*z* 1700 at 2 GHz acquisition rate). Accurate mass measurements were obtained through reference ion correction using reference masses at *m*/*z* 121.0509 (protonated purine) and 922.0098 [protonated hexakis(1H, 1H, 3H-tetrafluoropropoxy)phosphazene or HP-921] in positive ion mode. The compounds were confirmed in each spectrum.

The effluent from the LC column was directed from DAD into the ESI probe. The total run time for analysis was 25 min and detection wavelengths were 254, 280, and 330 nm.

### 2.2. Cell Culture

Human primary neurons and astrocytes, and mouse brain endothelial cell line (bEnd3, CRL-2299, ATCC) were used in this research. Human primary neurons and astrocytes were received from the National Scientific Medical Center, Nur-Sultan, Kazakhstan. The purity of astrocytic culture was assessed using immunofluorescence labeling of GFAP marker, the purity of neuronal culture was confirmed with immunofluorescence labeling of MAP2 marker. All cells were maintained in humidified 5% CO_2_ at 37 °C.

Only mycoplasma-free cell cultures were used in the present study. Delivered cell cultures were cultured in a quarantine room with a separate CO_2_ incubator and Class II safety cabinet one cell type at a time. After characterization of cell type and testing for contamination with bacteria, fungi and mycoplasma cells were transferred to the regular cell culture lab. On a regular schedule, once a month, or before performing the experiments, antibiotics were excluded from the medium, and cells were tested for mycoplasma using MycoFluor™ Mycoplasma Detection Kit (M7006, Invitrogen, Carlsbad, CA, USA).

Neurons were seeded on 96 well plates (density 5 × 10^3^/cm^2^). Cells were cultured in a complete neurobasal medium (21103049, Gibco, Carlsbad, CA, USA) containing 2% of B27 medium (17504044, Gibco) and 1% penicillin/streptomycin. Neurons were incubated with 10 μM cytosine arabinoside (C1768, Sigma, Taufkirchen, Germany) for 10 days. Before the experiments, cell culture was tested for purity by immunofluorescence labeling of MAP2 marker. To assess antioxidant properties of *Limonium gmelinii* extract, cells were starved and simultaneously pre-incubated with plant extract for 18 h followed by treatment with NMDA (M3262, Sigma), 100 μM for 30 min.

Primary human astrocytes and CECs were seeded to T-25 flasks (density 1 × 10^4^/cm^2^). The growth medium was changed every day, at 70–80% confluency the cells were re-seeded to the flasks or coverslips. All the experiments on astrocytes and CECs were performed at 3rd passages. To examine astrocytic phenotype prior experiments cells were tested for the GFAP marker. Astrocytes and bEnd3 cells were cultured in DMEM with 10% PBS and 1% antibiotic/antimycotic. To assess antioxidant properties of the plant extract, cells were preincubated with the extract (30 µg/mL) for 18 h with simultaneous starving. Astrocytes and CECs were exposed to 10 ng/mL TNF-α (T6674, Sigma) and 0.5 mM H_2_O_2_ (H1009, Sigma) for 60 min. After treatment, total ROS production, NADPH oxidase, P-selectin, and ERK 1/2 kinase activation were measured.

### 2.3. Fluorescent Intracellular ROS Assay

Overall, cellular ROS produced in the cells were measured using cell-permeable chloromethyl derivatives of 2′,7′-dichlorodihydrofluorescein diacetate (CM-H2DCFDA, C6827, Life Technologies, Carlsbad, CA, USA). Concurrently with treatment, cells were incubated with CM-H2DCFDA (2.5 µM) for 60 min. Fluorescent intensity of CM-H2DCFDA was measured using microplate reader Synergy H1 and was normalized to control.

### 2.4. Activation of NADP Oxidase Assay

The activation of NADPH-oxidase results in translocation of its cytosolic subunit (e.g., p47phox) to the cell membrane and membrane-associated subunits such as gp91phox [18,19]. We used confocal microscopy to quantify the co-localization of gp91phox and p47phox on the surface of the astrocytes. The protocol was previously described by Yang et al. [20]. Briefly, after the treatments, cells were fixed with 4% paraformaldehyde at 37 °C for 30 min., PBS containing 5% BSA was then applied to cells for 1 h to block nonspecific bindings. To label gp91phox at the cell surface, goat polyclonal anti-gp91phox (sc-5827, Santa Cruz Biotechnology, Dallas, TX, USA, 1:200 dilution) was added and incubated at 4 °C without permeabilization. To label p47phox, cells were then permeabilized with 0.1% Triton X-100 in PBS for 5 min. Rabbit polyclonal anti-p47phox (sc-14015, Santa Cruz Biotechnology, 1:200 dilution) in PBS with 1% BSA was then added and incubated at 4 °C overnight. This was followed by the fluorescent labeling with secondary antibodies (1:500 dilution) at room temperature for 1 h. The secondary antibodies for gp91phox and p47phox were Alexa 594 donkey anti-goat antibody (A-11058, Life Technologies), and Anti-Rabbit Alexa Fluor^®^ 488 secondary antibody (A-21441, Life Technologies), respectively. Secondary antibodies did not show immunostaining in the absence of the primary antibody (data not shown). Confocal immunofluorescence microscopy was performed with Carl Zeiss LSM700 confocal microscope. Confocal images were acquired with a 60X, numerical aperture 1.2 oil immersion objective lens for the co-localization studies between NADPH oxidase subunits, gp91phox and p47phox. To determine the spatial co-localization of labeled objects, Z-stack series at 1 μm spacing were captured. Background subtraction was done for all images before analysis. The colocalization indexes between two channels were calculated by normalizing the coincident intensity (yellow) to the total intensity of the corresponding channel as described previously. A total of 200 images were analyzed (50 images per group).

### 2.5. ERK 1/2 Kinases Assay

The ratios of total and phospho-ERK 1/2 kinases were measured in a cell lysate using Phospho/Total ERK 1/2 Whole Cell Lysate Kit (K15107D, Meso Scale Discovery, Rockville, MD, USA). All procedures were conducted following the manufacturer’s protocol. Cell lysates were collected using a lysis buffer provided with the kit. To discard cellular debris, lysates were centrifuged at 10,000× *g* for 10 min. Total and phospho-ERK 1/2 kinases were measured by electrochemiluminescence immunoassay in 96 well plates pre-coated with capture antibodies and incubated for 3 h. Then the plates were washed and incubated with a detection antibody for 1 h. After washing, a read buffer was added to each well and the plate was analyzed on MSD QuickPlex SQ 120. As a loading control, total protein in cell lysate was measured by BCA assay. Phospho-ERK/total ERK ratios in experimental groups were normalized to control.

### 2.6. Immunofluorescent Assay of P-Selectin Expression on the bEnd3 Cells Surface

Cells were fixed immediately after treatment using 3.7% paraformaldehyde solution for 30 min. To block non-specific binding, 5% BSA in PBS was applied to the cells for 1 h. Cellular surface P-selectin was labeled with its primary antibody (sc-25771, Santa Cruz, Dallas, TX, USA) without permeabilization at 4 °C overnight, followed by Goat anti-Rabbit IgG (H + L) Cross-Adsorbed Secondary Antibody Alexa Fluor™ 594 conjugate (A-11012, Invitrogen) labeling at room temperature for 1 h. To confirm the specificity of the primary antibodies, cells were labeled by secondary antibodies alone. Bright-field illumination and fluorescence microscopy were performed with fully motorized microscope Olympus IX83 and 40X, NA 0.95 objective. Images were acquired using a cooled CCD camera controlled with a computer and Meta Vue imaging software. The typical exposure time for fluorescence image acquisition was 400 msec. The background was subtracted for all images prior to analysis. P-selectin surface expression was quantified by calculating the ratio between integrated fluorescent intensity and the number of cells. The intensity was then normalized by the intensity of the labeled P-selectin in control cells (without any treatment). A total of 300 images were analyzed (50 images per group).

### 2.7. In Vivo Study

To assess neuroprotective properties of *L. gmelinii* extract in vivo, outbred male Wistar rats were used in this research. All procedures related to the animal studies were performed according to the protocols approved by the Ethics Committee of the Center for Life Sciences of Nazarbayev University (Registration number IORG 0006963). Animals were kept in normal vivarium conditions with a scheduled day/night cycle, at a temperature of 22–23 °C and received standard feed and drinking water ad libitum.

Ischemic stroke was induced in 16 rats aged 3–4 months through middle cerebral artery occlusion (MCAO). The advantage of the MCAO model is the similarity to the development of ischemic stroke (IS) in humans due to the ability to cause significant sizes of IS [21]. For obturation of the middle cerebral artery, silicone tipped 4/0 nylon monofilament (403734PK5Re, Doccol Corporation, Sharon, MA, USA) was used. A filament was submerged into an arteria carotis interna under the bipolar microscope to 17–20 mm depth. To make a focal zone of acute cerebral ischemia, monofilament was kept in this position for 2 h. IS was confirmed 24 h after occlusion by the presence of clinical symptoms (hemiparesis, hemianesthesia, hemianopsia, protrusion of the eyeball, unilateral ptosis). The syndrome of three “hemi” indicated an ischemic damage of the brain. Six animals were excluded from the experiments: four animals died during or immediately after the surgery, two animals did not show any signs of stroke. The remaining animals (weighing 280–300 g) were divided into 2 groups, 5 rats per group: (1) animals with IS and without any treatment (negative control), (2) animals with IS and treated with *Limonium Gmelinii* extract intragastrically (200 mg/kg for 28 days). Sensorimotor functions of rats were measured using a beam walking test a day before the MCAO (positive control), the next day, on the 7th, 14th, and 28th day after induction. After experiments, the animals were kept for further observation.

### 2.8. Evaluation of Sensorimotor Activity in Laboratory Animals

To analyze the locomotor function of rats, the front and hind legs’ motor coordination deficiency was measured by the Beam walk balance test (Open Science, Russia). All animals were trained to walk on a beam prior to the initial test. To compare locomotor function of healthy rats and animals with stroke the first beam walk test was performed before surgery. Afterwards, the tests were repeated on the 1st, 7th, 14th, and 28th day after surgery.

To start the test, an animal was placed in the dark at the beginning of the beam (wide part), after that, a bright light was turned on, compelling it to move forward to a narrowing path with the shelter at the end of the beam. Each passage of the rat was recorded on a video camera. All video records were analyzed using Realtimer software. To assess the motor deficiency of experimental rats, the number of paw puts on the lower board (error), the number of paw slips from the upper to the lower board (when the paw touches both boards) and the total number of steps taken by the rat from the beginning of the beam to the shelter were calculated. All calculations were done for front and hind paws separately. At least three attempts of tests for each animal were recorded and calculated. The percentage of sensorimotor deficiency was calculated using the following formula:


$$\frac{\mathrm{Error}+0.5\times \mathrm{Slipping}\times 100}{\mathrm{Total}\mathrm{number}\mathrm{of}\mathrm{steps}}$$

### 2.9. Statistical Analysis

For in vitro studies, data from at least three independent experiments were averaged, normalized to control, and presented as mean ± SD. The one-way analysis of variance (ANOVA) was used to investigate the effects of the plant extract on ROS formation, the activity of NADPH-oxidase, P-selectin expression, and phosphorylation of ERK 1/2 kinase. When the one-way ANOVA was significant, a post hoc test using Tukey’s pairwise comparison of means was used to reveal differences between various groups. For in vivo study, the data are also presented as mean ± SD. The differences between the two groups in each time point were evaluated using unpaired *t*-test. Values were considered significantly different at *p* ≤ 0.05. Statistical analyses were performed on the Stata 13; SigmaPlot^®^, version 11.0 software was used to graphically demonstrate the results.

## 3. Results

### 3.1. Chemical Characterization of the Plant Substance

**Figure 1:**
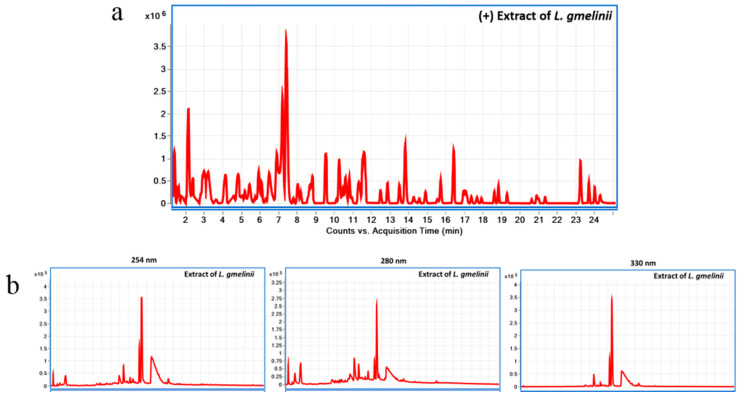
Chemical characterization of *L. gmelinii* root extract using Liquid chromatography diode array detector-quadrupole time-of-flight mass spectrometry (LC-DAD-QToF). LC-MS chromatogram of *L. gmelinii* extract in (+)ESI mode using LC-QToF (**a**); LC-UV chromatogram of *L. gmelinii* extract at 254, 280, and 330 nm using LC-DAD (**b**).

LC-MS chromatogram of *L. gmelinii* extract in (+)ESI mode using LC-QToF is presented in Figure 1a, and LC-UV chromatogram of the extract at 254, 280, and 330 nm using LC-DAD is presented in Figure 1b. The chemical composition of the plant substance is presented in the supplementary Table S1. In short, root extract of *Limonium gmelinii* contains a unique set of polyphenols in the form of phenolic acids (gallic and ellagic), flavonols, mainly aglycone myricetin and its glycoside forms, condensed tannins, the latter being represented by dimeric and oligomeric forms of flavan-3-ols. In the chemical study of the substance flavonols (myricetin, quercetin, kaempferol, and the new 3,4,5,3′,4′,6′-hexahydroxyoxyflavone), their glycosides (myricitrin, rutin, 3-β-galactosylquercetin, 3-β-galactosylmyricetin, new 3-α-galactopyranoside myricetin and 3-*O*-α-l-(2″-galloyl)-arabinopyranoside myricetin), pyrogallol are identified. The main monomeric flavan is (-)–epigallocatechin gallate. The new forms of flavan-3-ols, namely the 3,5,7,3′,4′,6′-hexahydroxyflavan, (-)–epigallocatechin-(4β→8)-(-)-3,5,7,3′,4′,6′–hexahydroxyflavan, and the (+)–gallocatechine–(4α→8)–[(-)–epigallocatechin]5–(4β→8)–(-)–epigallocatechin gallate, was found in the *L. gmelinii* root extract [22]. The quantitative content of tannins in the substance was 43.2%, flavonoids—15.23%, organic acids—6.36%, carbohydrates—5.44% [17]. Safety study of the substance conducted at the Test Center of the National Center of drugs, medical products, and medical equipment examination of the Ministry of Healthcare of the Republic of Kazakhstan has shown that it has no toxic effect on the animal organism and thus may be attributed to the group of non-toxic substances.

### 3.2. L. gmelinii Extract Attenuated NMDA-, H_2_O_2_, and TNF-α Induced Oxidative Response in Neurons, Astrocytes, and CECs

**Figure 2:**
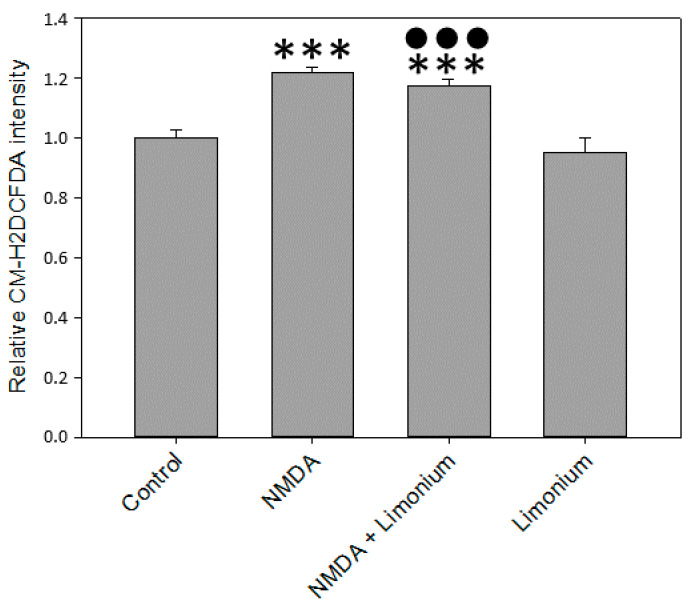
*L. gmelinii* extract inhibits ROS in human primary neurons exposed to NMDA. The level of ROS is represented by the relative intensity of CM-H2DCFDA normalized to the control group. ***—*p* ≤ 0.001 compared to the control; ●●●—*p* ≤ 0.001 compared to the group treated with NMDA.

Cellular ROS production in human primary neurons, astrocytes, and CECs was quantified with CM-H2DCFDA, a fluorescein dye commonly used as an indicator for ROS in cells. In agreement with previously published reports [8,23], our data indicated that in human primary neurons, 100 μM of NMDA increased CM-H2DCFDA fluorescence by 22% (*p* ≤ 0.001) as compared with untreated control (see Figure 2). At the same time, we observed a slight reduction of ROS (by 5%, *p* ≤ 0.001) in the group pre-treated with plant extract compared to the untreated cells.

**Figure 3:**
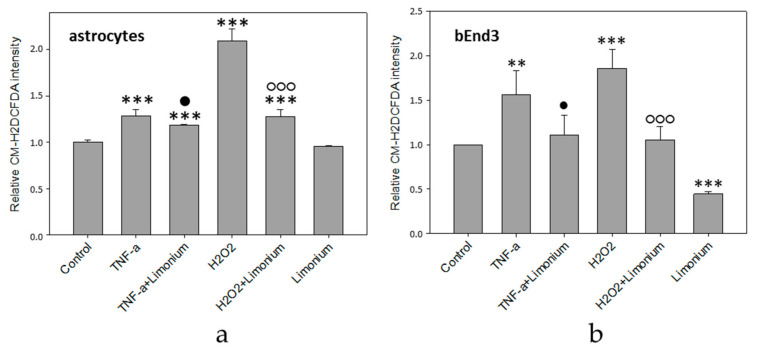
*L. gmelinii* extract reduces ROS in human primary astrocytes (**a**) and bEnd3 cells (**b**) treated with TNF-α and H_2_O_2_. The level of ROS is represented by the relative intensity of CM-H2DCFDA normalized to the control group. ***—*p* ≤ 0.001 compared to the control; **—*p* ≤ 0.01 compared to the control; ●—*p* ≤ 0.05 compared to the group treated with TNF-α; ○○○—*p* ≤ 0.001 compared to the group treated with H_2_O_2_.

The results of quantitative fluorescence analysis of the level of ROS generation in astrocytes and bEnd3 cells exposed to TNF-α are presented in Figure 3. As a positive control, we exposed the cells to H_2_O_2_, which is a known source of ROS. According to the data obtained, the treatment of astrocytes with TNF-α and H_2_O_2_ resulted in a 28 and 100% increase in the production of ROS, respectively, as compared to control (*p* ≤ 0.001) (see Figure 3a). Pre-treatment of the cells with the extract from *L. gmelinii* prevented the accumulation of ROS in astrocytes. As compared to cells that were exposed to TNF-α or H_2_O_2_ alone, ROS indicator was reduced by 10 and 73%, respectively (*p* ≤ 0.01, *p* ≤ 0.001). At the same time, the extract itself did not influence the generation of ROS in astrocytes, as there was no significant changes in the fluorescence intensity of the dye in this group. Similarly, the treatment of bEnd3 cells with TNF-α and H_2_O_2_ resulted in a 56 and 86% increase, respectively, in the production of ROS (*p* ≤ 0.01, *p* ≤ 0.001), while the presence of *L. gmelinii* extract completely attenuated accumulation of ROS in these cells (*p* ≤ 0.05, *p* ≤ 0.001). Moreover, the *Limonium* extract itself was capable of significantly reducing the generation of ROS in bEnd3 cells (*p* ≤ 0.001), (see Figure 3b).

It is worthy to note here that in bEnd3 cells treated with *L. gmelinii* extract ROS was significantly lower than in control cells, whereas in astrocytes it remained the same as in control condition. This difference might be attributed to the existence of slightly different ROS-dependent downstream pathways for CECs (bEnd3 cells in particular) and astrocytes that we have reported previously [24,25], albeit this is speculation.

### 3.3. Effect of L. gmelinii Extract on the Activity of NADPH Oxidase in Human Primary Astrocytes

**Figure 4:**
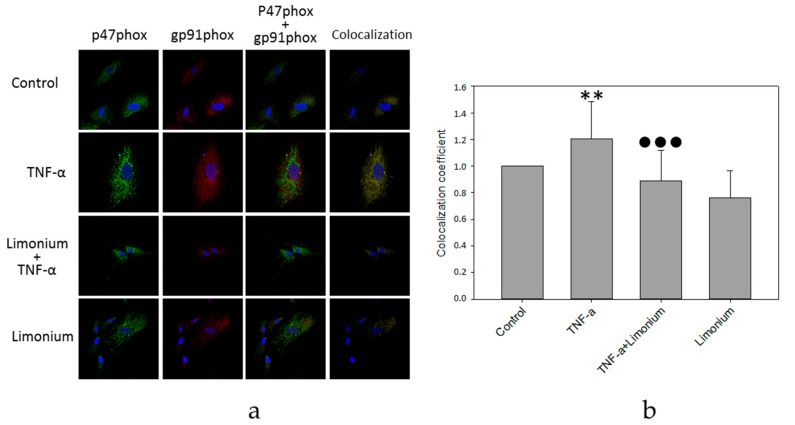
*L. gmelinii* extract suppresses activation of NADPH-oxidase induced by TNF-α in human primary astrocytes. NADPH-oxidase activity is represented by the coefficient of gp91phox and p47phox subunits co-localization (normalized to the control group). Confocal images (**a**), quantitative analysis (**b**). **—*p* ≤ 0.01 compared to the control; ●●●—*p* ≤ 0.001 compared to the group treated with TNF-α.

To further access antioxidant properties of *L. gmelinii,* we investigated the activity of NADPH oxidase in human primary astrocytes in the presence of TNF-α. NADPH-oxidase is a trans-membrane enzymatic complex comprising of two membrane-bound subunits (gp91-phox, p22-phox), three cytosolic subunits (p67-phox, p47-phox and p40-phox), and a low-molecular-weight G protein [26]. Several researchers have demonstrated that NADPH-oxidase actively participates in the production of ROS in astrocytes [24,27,28,29]. *Since* NADPH oxidase was activated by the assembly of the membrane-bound subunits with its cytosolic components, we tested the effects of *L. gmelinii* extract on co-localization of gp91phox and p47phox subunits by analyzing confocal images of double immunofluorescent-labeled gp91-phox and p47-phox in rat primary astrocytes (see Figure 4a,b).

As can be seen in Figure 4a, the number of yellow pixels in the combined images was minimal in the control group. In cells that were exposed to TNF-α, the number of yellow pixels increased markedly. In contrast, in the astrocytes, which were pre-incubated with polyphenol extract from *L. gmelinii*, and then with TNF-α, the level of yellow light glow on the combined images was much lower. Quantitative analysis showed that the level of co-localization of p47phox and gp91phox subunits of NADPH oxidase in astrocytes that were exposed to TNF-α increased by 20% (*p* ≤ 0.01), which indicates activation of this enzyme complex (see Figure 4b). For astrocytes that were pre-incubated with the extract, and then with TNF-α, the level of the co-localization of p47phox and gp91phox subunits was significantly lower (by 30%, *p* ≤ 0.001) compared to the cells that had only been exposed to TNF-α and remained at the control level suggesting either reversion or prevention of NADPH oxidase activation. The polyphenols extract itself had no significant effect on co-localization of the enzyme subunits.

### 3.4. Pretreatment with L. gmelinii Extract Suppressed Induced by TNF-α Pro-Inflammatory Responses in Astrocytes and CECs

**Figure 5:**
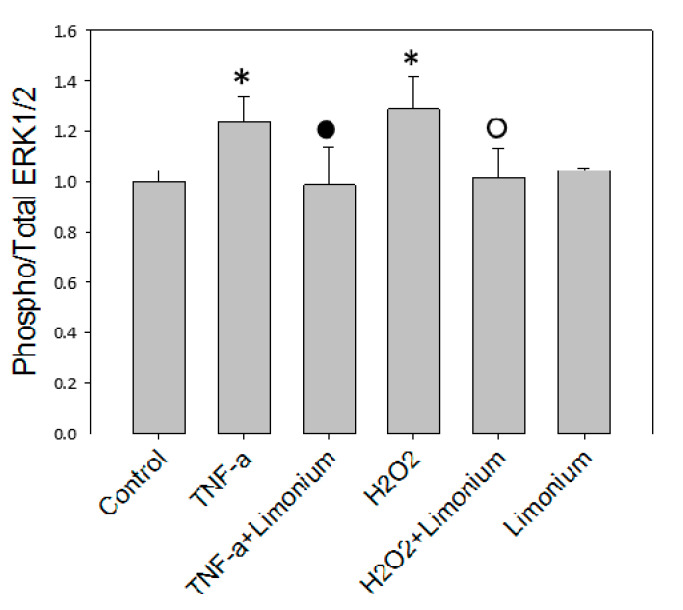
*L. gmelinii* extract suppresses activation of ERK 1/2 kinase induced by TNF-α in astrocytes. *—*p* ≤ 0.05 compared to the control; •—*p* ≤ 0.05 compared to the group treated with TNF-α; ○—*p* ≤ 0.05 compared to the group treated with H_2_O_2_.

To assess the effect of the extract on the activation of ERK 1/2 under the influence of TNF-α, an analysis of the level of phosphorylation of this enzyme was performed in human primary astrocytes by using MAP kinase assay. The results of the ERK 1/2 protein kinase activity shown in Figure 5 demonstrated that the level of phosphorylation of ERK 1/2 was significantly higher (by 23.5%, *p* ≤ 0.05) in cells that were exposed to TNF-α compared to the control. If cells were pretreated with the extract phosphorylated enzyme content did not increase as much as in a TNF-α group, this suggested either reversion or prevention of ERK 1/2 activation. In this group, the amount of phospho-ERK 1/2 almost reached the control values. The extract itself did not affect the activity of the enzyme. The level of its phosphorylation in cells that were incubated only with the extract did not significantly differ from the control one.

**Figure 6:**
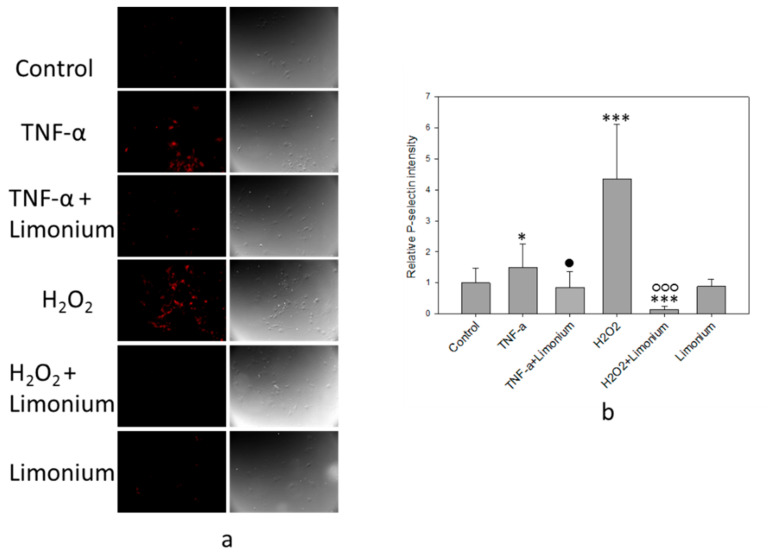
*L. gmelinii* extract suppresses TNF-α and H_2_O_2_-induced expression of P-selectin on the cell surface. Fluorescent images of P-selectin labeled bEnd3cells (**a**); relative intensity of P-selectin in the bEnd3 (**b**). ***—*p* ≤ 0.001 compared to the *control*; *—*p* ≤ 0.05 compared to the *control*; •—*p* ≤ 0.05 compared to the cells treated only with TNF-α; ○○○—compared to the cells treated only with H_2_O_2_.

The results of immunofluorescence analysis of P-selectin mobilization are presented in Figure 6. Under the influence of TNF-α, the fluorescence intensity of the labeled P-selectin increased (see Figure 6a, colored red) as compared with the control, and in cells that had been pretreated with the extract, a decrease in the emission of the labeled P-selectin was observed. Quantitative analysis of the P-selectin content on the cell surface showed that, upon exposure to TNF-α, the P-selectin level increased by 50% (*p* ≤ 0.05) compared to the control. On the contrary, pre-incubation of the cells with plant extract prevented overexpression of the P-selectin on the surface of the cells (by 65%, *p* ≤ 0.05, compared to TNF-α only treated group). In cells that were incubated only with plant extract, the P-selectin content on the surface did not differ from the control level (see Figure 6b).

We have previously reported that phosphorylation of ERK 1/2 in astrocytes and mobilization of P-selectin in CECs could be induced by ROS [25,30]. As a positive control, in the present study, we have exposed the cells to H_2_O_2_ (see Figure 5 and Figure 6). In agreement with our earlier studies, exposure of the cells to H_2_O_2_ resulted in a 32% increase of Phospho/Total ERK 1/2 ratio (*p* ≤ 0.05, compared to control) in human primary astrocytes and 4 fold increase of the P-selectin levels in CECs (*p* ≤ 0.001, compared to control), while pretreatment with the plant extract either prevented or reversed phosphorylation of ERK 1/2 (*p* ≤ 0.001, compared to H_2_O_2_) and overexpression of P-selectin on the surface of CECs (*p* ≤ 0.001, compared to H_2_O_2_).

### 3.5. L. gmelinii Extract Improved Motor Activity in Rats with MCAO

As mentioned above, to study the neuroprotective effect of the extract from *L. gmelinii* on a model of laboratory animals, ischemic stroke was induced in male Wistar rats by occlusion of the middle cerebral artery (MCAO). The next day after the induction of the stroke, the animals started to receive an extract from the *L. gmelinii* at 200 mg/kg, intragastrically for 28 days.

**Figure 7:**
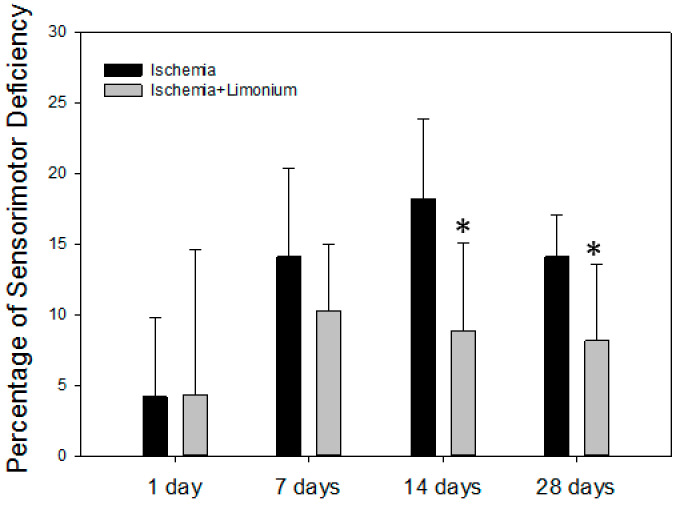
*L. gmelinii* extract improves motor coordination of rats with induced ischemic stroke after 14 and 28 days of treatment. The percentage of motor deficiency was calculated and normalized to the control trials (before surgery, represented as a 0 point). *—*p* ≤ 0.05 compared to the animals with ischemia only.

In all the operated animals, in the first hours after waking up and on the first day of the postoperative period, development of neurological deficiency was observed, manifested by the development of lethargy and slowed movements, unilateral ptosis of the eye on the affected side, paresis or paralysis of the right anterior and/or hind paws, tremor, hemianesthesia of the affected side, suppression of appetite, dysfunction of the regulation of defecation and urination, and deterioration of sensorimotor activity (see Figure 7).

On the seventh day after the beginning of the experiment (MCAO), both groups of the animals showed an increase in sensorimotor disturbances: by 14.1% in the control group and by 10.3% in the treatment group. In control, a sensorimotor deficiency was increased on the 14th day (by 18.2%) and was still present on the 28th day (14.1%). In contrast, we did not observe negative dynamics on days 14 and 28 in animals treated with plant extract; moreover, motor activity was significantly improved in this group (by 6.8 and 4.5% compared to untreated control, *p* ≤ 0.05).

## 4. Discussion

This study is aimed at investigating the antioxidant and neuroprotective properties of polyphenols from the *L. gmelinii* plant. The results of the study of the influence of polyphenols extract from *L. gmelinii* on the generation of ROS show that this extract at a dosage of 30 μg/mL can partially protect primary human neurons from NMDA-induced oxidative stress and significantly attenuates TNF-α and H_2_O_2_ induced accumulation of ROS in human primary astrocytes, and mouse CECs. The antioxidant effect of the extract is likely associated with a high content of flavonoids (15.23%) and tannins (43.2%). It has been demonstrated that flavonoids and tannins are highly effective radical scavengers due to the catechol ring of flavonoids and trigalloyl moieties of gallic acid–based compounds [31,32].

However, as mentioned above, this extract has a complex multicomponent composition, so it is a reasonable assumption that it can also have a modulating effect directed at various enzyme systems of the cell. For example, there is evidence that flavonoids and their in vivo metabolites act not only as straight hydrogen-donating antioxidants, but also can directly attune protein kinase and lipid kinase signaling pathways in the cells. For example, it has been demonstrated that flavonoids and their metabolites regulate PI 3-kinase, Akt/PKB, tyrosine kinases, PKC, and MAP kinase signaling cascades [33,34].

Considering the chemical composition of root extract from *L. gmelinii*, it is logical to assume that it has a direct effect on enzymatic complexes producing oxygen radicals. As is known, NADPH oxidase is one of the most important sources of ROS in astrocytes and CECs [27,28,29]. In connection with the foregoing, we conducted a study of the effect of this extract on the activation of NADPH oxidase in human primary astrocytes. Based on the obtained data, it can be concluded that TNF-α induces the assembly of cytoplasmic and membrane subunits of NADPH oxidase of astrocytes and activates this enzyme complex. Polyphenols extract from *L. gmelinii* inhibits activation of NADPH oxidase, and therefore, probably reduces the level of synthesis of ROS in astrocytes.

One of the most important systems in astrocytes and other cell types, which are involved in the development of inflammatory processes implicated in the pathogenesis of neurodegenerative disorders and ischemic brain injury, are signaling pathways mediated by MAP kinases such as p38, JNK, and ERK 1/2. Several studies have demonstrated that Aβ oligomers and ROS can initiate the downstream mitogen-activated protein kinase/extracellular signal-regulated kinase (MAPK/ERK) pathway in astrocytes [24,25,35,36]. Transcriptome analysis of gene expression suggested the involvement of the MAPK signaling pathway in human temporal lobe epilepsy and hypothermia [37,38]. These protein kinases are involved in the activation of transcription factors controlling the expression of pro-inflammatory cytokines, and through the activation of various pathways regulate the assembly of NADPH oxidase and the formation of ROS [24,39].Substances that inhibit the activity of these enzymes can have a great therapeutic potential in the treatment of diseases of the central nervous system, including post-stroke states. In this connection, we studied the effect of polyphenols extract from *L. gmelinii* on the activity of ERK 1/2 under the influence of TNF-α. It was identified that TNF-α activates ERK 1/2, which resulted in an increase of phosphorylated protein content in cells. Polyphenols extract from *L. gmelinii* reduced the activity of the studied protein kinase and thereby blocked the toxic effect of TNF-α.

The pro-inflammatory effect of TNF-α is not limited to the generation of ROS, activation of NADPH oxidase and phosphorylation of ERK 1/2. TNF-α is involved in the development of inflammatory processes that occur in ischemic or neurodegenerative brain damage due to many other mechanisms. For instance, TNF-α can induce the transmigration of blood cells through the blood-brain barrier into the brain parenchyma. Transmigration of immunocompetent blood cells is one of the earliest stages of the development of inflammation in the brain parenchyma [40]. It is regulated by cell adhesion receptors expressed on the surface of CECs, such as selectins [30,40]. It is known that ROS and TNF-α lead to the activation of CECs, followed by the mobilization of P-selectin on the luminal surface of the endothelium [30,41]. In connection with this, one of the tasks of this study was to investigate the effect of polyphenols extract from *L. gmelinii* on the TNF-α-induced mobilization of P-selectin. In our study, ROS (H_2_O_2_) and TNF-α promoted endothelial surface receptor P-selectin expression, while pre-treatment with the extract of *L. gmelinii* significantly reduced the fluorescence of P-selectin. Based on the obtained data, it can be concluded that polyphenols extract from *L. gmelinii* inhibited the mobilization of P-selectin induced by the cytokine TNF-α. As mentioned earlier, mobilization of P-selectin on the surface of endothelial cells is one of the earliest mechanisms triggering the transmigration of leukocytes and monocytes through the BBB in the development of neuroinflammation. Substances that can inhibit the redisposition of P-selectin from the cytoplasm to the surface of CECs can be used to develop methods for brain disorders therapy.

To verify the neuroprotective properties of *L. gmelinii* extract in vivo, we evaluated its therapeutic potential on an animal model of ischemic brain injury. Ischemic stroke is the third cause of death after heart disease and cancer, and the leading cause of long-term disability in aging adults in most countries [42], and 85% of stroke cases are ischemic in nature and caused by occlusion of blood vessels or arteriole ends, which results in blood supply deprivation to the brain. Although early restoration of blood flow is critical to save a patient’s live, reperfusion itself causes further damage to cerebral tissue [43]. During reperfusion, blood returns to an infarcted brain area carrying leukocytes. When reaching the microvasculature in the damaged area, these leukocytes transmigrate into the brain parenchyma in response to the cell adhesion molecules expressed on the surface of cerebral endothelial cells (CECs) [44]. Here, leukocytes release reactive oxygen species (ROS) and further inflammatory mediators [45]. In line with data demonstrating the critical role of edema in stroke-associated morbidity and mortality, several studies have shown that post-ischemic edema in the brain is associated with brain fluid homeostasis and strictly regulated by aquaporin 4 (AQP4) channels, which are mainly expressed in perivascular astrocytic end-feet, and targeting AQP4 could be a useful therapeutic approach for treating brain ischemia and edema [46,47,48,49]. Moreover, some evidence exists showing the role of polyphenols, such as curcumin, pinocembrin, resveratrol, and quercetin, in modulating the activity of some aquaporin (AQP) isoforms, which could also contribute to neuroprotective effects exerted by plant polyphenols [50,51]. In our study we have found that motor activity significantly improved in animals subjected to MCAO and treated with *L. gmelinii* extract as compared to untreated controls, and we suggest that the therapeutic potential of plant extract is associated with its antioxidant, anti-inflammatory, and modulatory properties described above.

## 5. Conclusions

In conclusion, the extract from roots and rhizomes of *Limonium gmelinii* protects neurons, astrocytes, and cerebral endothelial cells from oxidative and inflammatory responses in vitro, improves motor functions after ischemic stroke in vivo, and could be recommended for further pre-clinical and clinical studies.

## Data Availability

Data is contained within the article.

## Supplementary Materials

The following are available online at https://www.mdpi.com/article/10.3390/antiox10111814/s1, Table S1: Non-targeted analysis of extracts of *L. gmelinii* root in (+)ESI mode using LC-QToF.

## References

[1] Kim G.H., Kim J.E., Rhie S.J., Yoon S. (2015). The Role of Oxidative Stress in Neurodegenerative Diseases. Exp. Neurobiol..

[2] Redza-Dutordoir M., Averill-Bates D.A. (2016). Activation of apoptosis signalling pathways by reactive oxygen species. Biochim. Biophys. Acta BBA-Mol. Cell Res..

[3] Carvalho C., Moreira P.I. (2018). Oxidative Stress: A Major Player in Cerebrovascular Alterations Associated to Neurodegenerative Events. Front. Physiol..

[4] Chen Y., Qin C., Huang J., Tang X., Liu C., Huang K., Xu J., Guo G., Tong A., Zhou L. (2020). The role of astrocytes in oxidative stress of central nervous system: A mixed blessing. Cell Prolif..

[5] Chen H., Yoshioka H., Kim G.S., Jung J.E., Okami N., Sakata H., Maier C.M., Narasimhan P., Goeders C.E., Chan P.H. (2011). Oxidative stress in ischemic brain damage: Mechanisms of cell death and potential molecular targets for neuroprotection. Antioxid. Redox. Signal.

[6] Lee K.H., Cha M., Lee B.H. (2020). Neuroprotective Effect of Antioxidants in the Brain. Int. J. Mol. Sci..

[7] Simonyi A., Wang Q., Miller R.L., Yusof M., Shelat P.B., Sun A.Y., Sun G.Y. (2005). Polyphenols in cerebral ischemia: Novel targets for neuroprotection. Mol. Neurobiol..

[8] Chuang D.Y., Chan M.H., Zong Y., Sheng W., He Y., Jiang J.H., Simonyi A., Gu Z., Fritsche K.L., Cui J. (2013). Magnolia polyphenols attenuate oxidative and inflammatory responses in neurons and microglial cells. J. Neuroinflammation.

[9] Liu X., Wang Z., Wang P., Yu B., Liu Y., Xue Y. (2013). Green tea polyphenols alleviate early BBB damage during experimental focal cerebral ischemia through regulating tight junctions and PKCalpha signaling. BMC Complement. Altern. Med..

[10] Panickar K.S., Jang S. (2013). Dietary and plant polyphenols exert neuroprotective effects and improve cognitive function in cerebral ischemia. Recent Pat. Food Nutr. Agric..

[11] Abib R.T., Quincozes-Santos A., Nardin P., Wofchuk S.T., Perry M.L., Goncalves C.A., Gottfried C. (2008). Epicatechin gallate increases glutamate uptake and S100B secretion in C6 cell lineage. Mol. Cell Biochem..

[12] Mahler A., Mandel S., Lorenz M., Ruegg U., Wanker E.E., Boschmann M., Paul F. (2013). Epigallocatechin-3-gallate: A useful, effective and safe clinical approach for targeted prevention and individualised treatment of neurological diseases?. EPMA J..

[13] Manach C., Scalbert A., Morand C., Remesy C., Jimenez L. (2004). Polyphenols: Food sources and bioavailability. Am. J. Clin. Nutr..

[14] Shalakhmetova T., Zhusupova G., Askarova S. (2010). Antioxidative and hepatoprotective properties of phyto medicine extracted from *Limonium gmelinii*. Int. J. Biol. Chem..

[15] Maryam M., Lack H.W., Maria L., Hossein A. (2017). The discovery, naming and typification of *Limonium gmelini* (*Plumbaginaceae*). Willdenowia.

[16] Askarova S., Sun Z., Sun G.Y., Meininger G.A., Lee J.C. (2013). Amyloid-β peptide on sialyl-Lewis(X)-selectin-mediated membrane tether mechanics at the cerebral endothelial cell surface. PLoS ONE.

[17] Abduakhitovich A.Z., Dyusembaevich R.K., Eventaevna Z.G. Method for obtaining a total polyphenolic complex from the roots of *Limonium gmelinii*. RK Patent No. 14418 from 15.10.2007. bul. No. 10 (rus.). https://kzpatents.com/5-14418-sposob-polucheniya-summarnogo-polifenolnogo-kompleksa-iz-kornejj-kermeka-gmelina.html.

[18] Samuelson D.J., Powell M.B., Lluria-Prevatt M., Romagnolo D.F. (2001). Transcriptional activation of the gp91phox NADPH oxidase subunit by TPA in HL-60 cells. J. Leukoc. Biol..

[19] DeLeo F.R., Allen L.A.H., Apicella M., Nauseef W.M. (1999). NADPH oxidase activation and assembly during phagocytosis. J. Immunol..

[20] Yang X.G., Askarova S., Sheng W.W., Chen J.K., Sun A.Y., Sun G.Y., Yao G., Lee J.C.M. (2011). Low Energy Laser Light (632.8 nm) Suppresses Amyloid-Beta Peptide-Induced Oxidative and Inflammatory Responses in Astrocytes. Biophys. J..

[21] Uluç K., Miranpuri A., Kujoth G.C., Aktüre E., Başkaya M.K. (2011). Focal cerebral ischemia model by endovascular suture occlusion of the middle cerebral artery in the rat. J. Vis. Exp..

[22] Zhusupova G.E., Abil’kaeva S.A. (2006). Dimeric prodelphinidins from *Limonium gmelinii* roots. III. Chem. Nat. Compd..

[23] Shelat P., Chalimoniuk M., Wang J.-H., Strosznajder J., Lee J., Simonyi A., Sun G. (2008). Amyloid beta peptide and NMDA induce ROS from NADPH oxidase and AA release from cytosolic phospholipase A. J. Neurochem..

[24] Askarova S., Yang X., Sheng W., Sun G.Y., Lee J.C.M. (2011). Role of Aβ-receptor for advanced glycation endproducts interaction in oxidative stress and cytosolic phospholipase A₂ activation in astrocytes and cerebral endothelial cells. Neuroscience.

[25] Tsoy A., Saliev T., Abzhanova E., Turgambayeva A., Kaiyrlykyzy A., Akishev M., Saparbayev S., Umbayev B., Askarova S. (2019). The Effects of Mobile Phone Radiofrequency Electromagnetic Fields on β-Amyloid-Induced Oxidative Stress in Human and Rat Primary Astrocytes. Neuroscience.

[26] Babior B.M. (1999). NADPH Oxidase: An Update. Blood.

[27] Qing L., Jiu-Hong K., Rong-Liang Z. (2005). NADPH oxidase produces reactive oxygen species and maintains survival of rat astrocytes. Cell Biochem. Funct..

[28] Zhu D., Hu C., Sheng W., Tan K.S., Haidekker M.A., Sun A.Y., Sun G.Y., Lee J.C.-M. (2009). NAD(P)H oxidase-mediated reactive oxygen species production alters astrocyte membrane molecular order via phospholipase A2. Biochem J..

[29] Sheng W.S., Hu S., Feng A., Rock R.B. (2013). Reactive oxygen species from human astrocytes induced functional impairment and oxidative damage. Neurochem. Res..

[30] Tsoy A., Shalakhmetova T., Umbayev B., Askarova S. (2014). Role of ros in aβ42 mediated cell surface p-selectin expression and actin polymerization. Neurol. Asia.

[31] Maisetta G., Batoni G., Caboni P., Esin S., Rinaldi A.C., Zucca P. (2019). Tannin profile, antioxidant properties, and antimicrobial activity of extracts from two Mediterranean species of parasitic plant Cytinus. BMC Complementary Altern. Med..

[32] Ricci A., Olejar K.J., Parpinello G.P., Mattioli A.U., Teslić N., Kilmartin P.A., Versari A. (2016). Antioxidant activity of commercial food grade tannins exemplified in a wine model. Food Addit. Contam. Part A.

[33] Williams R.J., Spencer J.P., Rice-Evans C. (2004). Flavonoids: Antioxidants or signalling molecules?. Free Radic. Biol. Med..

[34] Mansuri M.L., Parihar P., Solanki I., Parihar M.S. (2014). Flavonoids in modulation of cell survival signalling pathways. Genes Nutr..

[35] Zhu X., Lee H.-G., Raina A.K., Perry G., Smith M.A. (2002). The Role of Mitogen-Activated Protein Kinase Pathways in Alzheimer’s Disease. Neurosignals.

[36] Son Y., Kim S., Chung H.T., Pae H.O. (2013). Reactive oxygen species in the activation of MAP kinases. Methods Enzym..

[37] Salman M.M., Kitchen P., Woodroofe M.N., Bill R.M., Conner A.C., Heath P.R., Conner M.T. (2017). Transcriptome Analysis of Gene Expression Provides New Insights into the Effect of Mild Therapeutic Hypothermia on Primary Human Cortical Astrocytes Cultured under Hypoxia. Front. Cell Neurosci..

[38] Salman M.M., Sheilabi M.A., Bhattacharyya D., Kitchen P., Conner A.C., Bill R.M., Woodroofe M.N., Conner M.T., Princivalle A.P. (2017). Transcriptome analysis suggests a role for the differential expression of cerebral aquaporins and the MAPK signalling pathway in human temporal lobe epilepsy. Eur. J. Neurosci..

[39] Keshari R.S., Verma A., Barthwal M.K., Dikshit M. (2013). Reactive oxygen species-induced activation of ERK and p38 MAPK mediates PMA-induced NETs release from human neutrophils. J. Cell Biochem..

[40] McEver R.P., Zhu C. (2010). Rolling cell adhesion. Annu. Rev. Cell Dev. Biol..

[41] Liu Y.J., Guo D.W., Tian L., Shang D.S., Zhao W.D., Li B., Fang W.G., Zhu L., Chen Y.H. (2010). Peripheral T cells derived from Alzheimer’s disease patients overexpress CXCR2 contributing to its transendothelial migration, which is microglial TNF-alpha-dependent. Neurobiol. Aging.

[42] Feigin V.L., Forouzanfar M.H., Krishnamurthi R., Mensah G.A., Connor M., Bennett D.A., Moran A.E., Sacco R.L., Anderson L., Truelsen T. (2014). Global and regional burden of stroke during 1990-2010: Findings from the Global Burden of Disease Study 2010. Lancet.

[43] Wu L., Xiong X., Wu X., Ye Y., Jian Z., Zhi Z., Gu L. (2020). Targeting Oxidative Stress and Inflammation to Prevent Ischemia-Reperfusion Injury. Front. Mol. Neurosci..

[44] Frijns C.J.M., Kappelle L.J. (2002). Inflammatory Cell Adhesion Molecules in Ischemic Cerebrovascular Disease. Stroke.

[45] Savman K., Heyes M.P., Svedin P., Karlsson A. (2013). Microglia/macrophage-derived inflammatory mediators galectin-3 and quinolinic acid are elevated in cerebrospinal fluid from newborn infants after birth asphyxia. Transl. Stroke Res..

[46] Kitchen P., Salman M.M., Halsey A.M., Clarke-Bland C., MacDonald J.A., Ishida H., Vogel H.J., Almutiri S., Logan A., Kreida S. (2020). Targeting Aquaporin-4 Subcellular Localization to Treat Central Nervous System Edema. Cell.

[47] Sylvain N.J., Salman M.M., Pushie M.J., Hou H., Meher V., Herlo R., Peeling L., Kelly M.E. (2021). The effects of trifluoperazine on brain edema, aquaporin-4 expression and metabolic markers during the acute phase of stroke using photothrombotic mouse model. Biochim. Biophys. Acta Biomembr..

[48] Salman M.M., Kitchen P., Halsey A., Wang M.X., Tornroth-Horsefield S., Conner A.C., Badaut J., Iliff J.J., Bill R.M. (2021). Emerging roles for dynamic aquaporin-4 subcellular relocalization in CNS water homeostasis. Brain.

[49] Salman M.M., Kitchen P., Iliff J.J., Bill R.M. (2021). Aquaporin 4 and glymphatic flow have central roles in brain fluid homeostasis. Nat. Rev. Neurosci..

[50] Fiorentini D., Zambonin L., Vieceli Dalla Sega F., Hrelia S. (2015). Polyphenols as Modulators of Aquaporin Family in Health and Disease. Oxidative Med. Cell. Longev..

[51] Tesse A., Grossini E., Tamma G., Brenner C., Portincasa P., Marinelli R.A., Calamita G. (2018). Aquaporins as Targets of Dietary Bioactive Phytocompounds. Front. Mol. Biosci..

