# Supplementary material for: Plant Extract of Limonium gmelinii Attenuates Oxidative Responses in Neurons, Astrocytes, and Cerebral Endothelial Cells In Vitro and Improves Motor Functions of Rats after Middle Cerebral Artery Occlusion
Source: Antioxidants (Basel). 2021 Nov 15;10(11):1814. doi: 10.3390/antiox10111814 (PMC8614848; doi:10.3390/antiox10111814)
Supplement: Supplementary file 1 [file antioxidants-10-01814-s001.zip › antioxidants-1431183-supplementary.pdf]

Table S1. Non-targeted analysis of extracts of *L. gmelinii* root in (+)ESI mode using LC-QToF.

#	RT (min)	Compound Name	Formula	Mass	$[M+H]^+$	Fragment Ions
1	11.5	Myricetin 7-rhamnoside/Myricetin 3-*O*-α-L-rhamnopyranoside (Myricitrin)	$C_{21}H_{20}O_{12}$	464.0955	465.1025 (465.1028)	319.0439
2	11.6					
3	9.93	Myricetin 3-galactoside (Gmelinoside I)/ Myricetin 3-β-D-sorboside/Myricetin 3-glucoside	$C_{21}H_{20}O_{13}$	480.0904	481.0975 (481.0977)	319.0436
4	10.26					
5	10.45					
6	12.41					
7	14.6	2',4',5,5',7-Pentahydroxyflavonol/5'-Hydroxymorin	$C_{15}H_{10}O_8$	318.0376	319.0439 (319.0448)	-
8	9.63	Myricetin 7-(6''-galloylglucoside)	$C_{28}H_{24}O_{17}$	632.1013	633.1081 (633.1086)	319.0431 153.0131
9	10.8	Myricetin 3-xyloside	$C_{20}H_{18}O_{12}$	450.0798	451.0869 (451.0871)	319.0433
10	11.3					
11	12.1	Laricitrin 7-glucoside	$C_{22}H_{22}O_{13}$	494.106	495.1121 (495.1133)	
12	11.8/12.2	Quercetin glucoside	$C_{21}H_{19}O_{12}$	464.0955	465.1021 (465.1028)	303.0509
13	10.9	Quercetin 3-(2''-galloylgalactoside	$C_{28}H_{24}O_{16}$	616.1064	617.1131 (617.1137)	303.0441
14	12.3	Myricetin-3-*O*-[3,4,5-Trihydroxybenzoyl-(→2)-α-L-arabinopyranoside]	$C_{27}H_{22}O_{16}$	602.0908	603.0975 (603.0981)	319.0429 153.0164
15	9.2	Epigallocatechin gallate/Gallo epicatechin gallate	$C_{22}H_{18}O_{11}$	458.0849	459.0927 (459.0922)	139.0396
16	8.8	Epicatechin/Catechin	$C_{15}H_{14}O_6$	290.079	291.0857 (291.0863)	139.0391
17	4.07	Gallocatechin/Epigallocatechin/ 3,5,7,3′,4′,6′-hexahydroxyflavan	$C_{15}H_{14}O_7$	306.074	307.0811 (307.0812)	139.0357
18	4.5					
19	5.4					
20	6.45					
21	3.8	Phenylalanine	$C_9H_{11}NO_2$	165.079	166.0861 (166.0859)	103.0536 77.0385
22	18.5	Dihydrosyringetin	$C_{17}H_{15}O_8$	346.0689	347.0755 (347.0761)	-
23	9.02	Aromadendrene	$C_{15}H_{12}O_6$	288.0634	289.0701 (289.0707)	-

24	3.09	Gallic Acid	$C_7H_4O_5$	168.0059	169.0129 (169.0131)	125.0258
25	13.4	Quercitrin	$C_{21}H_{20}O_{11}$	448.1006	449.1071 (449.1078)	303.04951
26	3.9	(-)-Epigallocatechin-(4β→8)-(-)-3,5,7-3′,4′,6′-hexahydroxyflavan	$C_{30}H_{26}O_{14}$	610.1323	611.1401 (611.1395)	307.0799 139.0351
27	17.5	Kaempferol	$C_{15}H_{10}O_6$	286.0477	287.0545 (287.055)	-
28	6.6	(-)-Epigallocatechin-(4β→8)-(-)-epigallocatechin-3-*O*-gallate	$C_{37}H_{30}O_{18}$	762.1432	763.1499 (763.1505)	307.0801, 153.0212 139.0404
29	15.5	Limoniumin A	$C_{26}H_{19}NO_7$	457.1162	458.1231 (458.1234)	287.0404
30	16.79	Limoniumin B	$C_{27}H_{21}NO_7$	471.1318	472.1385 (472.1391)	
31	17.1	Limoniumin F/H/I/Cannabisin C	$C_{35}H_{34}N_2O_8$	610.2315	611.2398 (611.2388)	
32	17.3					
33	17.9					
34	15.77	Cannabisin B	$C_{34}H_{32}N_2O_8$	596.2159	597.2245 (597.2231)	295.0478
35	15.52	Cannabisin A	$C_{34}H_{30}N_2O_8$	594.2002	595.2071 (595.2075)	
36	18.6	Cannabisin D/F	$C_{36}H_{36}N_2O_8$	624.2472	625.2535 (625.2544)	
37	18.8					
38	20.9					
39	16.4	*N-trans*-feruloyl tyramine	$C_{18}H_{19}NO_4$	313.1314	314.1381 (314.1381)	177.0448 145.0183 117.0241
40	19.3	Thoreliamide B	$C_{28}H_{29}NO_8$	507.1893	508.1956 (508.1966)	
41	15.65	3,3′-Demethyl-heliotropamide	$C_{34}H_{32}N_2O_8$	596.2159	597.2223 (597.2231)	295.0460 187.0261 153.0057
42	5.9	(+)–gallocatechine–(4α→8)–[(-)–epigallocatechin]5–(4β→8)–(-)–epigallocatechin gallate	$C_{52}H_{42}O_{25}$	1066.2015	1067.2088	153.0089 139.0293
43	8.19					
